# Old age mental health services in Southern Balkans: Features, geospatial distribution, current needs, and future perspectives

**DOI:** 10.1192/j.eurpsy.2020.85

**Published:** 2020-09-14

**Authors:** P. Alexopoulos, A. Novotni, G. Novotni, T. Vorvolakos, A. Vratsista, A. Konsta, S. Kaprinis, A. Konstantinou, K. Bonotis, E. Katirtzoglou, K. Siarkos, E. S. Bekri, I. Kokkoris, A. Como, R. Gournellis, D. S. Stoyanov, A. Politis

**Affiliations:** 1 Department of Psychiatry, Patras University General Hospital, Faculty of Medicine, School of Health Sciences, University of Patras, Patras, Greece; 2 Department of Psychiatry and Psychotherapy, Klinikum rechts der Isar, Faculty of Medicine, Technische. Technische Universität München, Munich, Germany; 3 University Clinic of Psychiatry, Medical Faculty, University “Ss Cyril and Methodius”, Skopje, North Macedonia; 4 University Clinic of Neurology, Medical Faculty, University “Ss Cyril and Methodius”, Skopje, North Macedonia; 5 Department of Psychiatry, Alexandroupolis University General Hospital, Faculty of Medicine, School of Health Sciences, Democritus University of Thrace, Alexandroupolis, Greece; 6 Department of Psychiatry, Arta General Hospital, Arta, Greece; 7 1^st^ Department of Psychiatry, “Papageorgiou” General Hospital, Faculty of Medicine, Aristotle University of Thessaloniki, Thessaloniki, Greece; 8 3^rd^ Department of Psychiatry. Psychiatric Hospital of Thessaloniki, Faculty of Medicine, Aristotle University of Thessaloniki, Thessaloniki, Greece; 9 Department of Psychiatry, Larissa University General Hospital, Faculty of Medicine, University of Thessaly, Larisa, Greece; 10 1st Department of Psychiatry, “Eginition” University Hospital, School of Medicine, National and Kapodistrian University of Athens, Athens, Greece; 11 Environmental Engineering Laboratory, Department of Civil Engineering, School of Engineering, University of Patras, Patras, Greece; 12 Division of Plant Biology, Department of Biology, School of Basic Sciences, University of Patras, Patras, Greece; 13 Psychiatry Division, Department of Neuroscience, Tirana University Hospital Center “Mother Teresa”, Tirana Medical University, Tirana, Albania; 14 2nd Department of Psychiatry, “Attikon” University General Hospital, School of Medicine, National and Kapodistrian University of Athens, Athens, Greece; 15 Department of Psychiatry and Medical Psychology, Faculty of Medicine, Medical University of Plovdiv, Plovdiv, Bulgaria; 16 Department of Psychiatry, Division of Geriatric Psychiatry and Neuropsychiatry, Johns Hopkins Medical School, Baltimore, USA

**Keywords:** liaison, in- and outpatient psychogeriatric services, research and didactic activity, telepsychiatry

## Abstract

**Background:**

Healthcare services are increasingly confronted with challenges related to old age mental disorders. The survey aimed to provide an overview of existing psychogeriatric services in Albania, Bulgaria, Greece, and North Macedonia.

**Methods:**

After identification of psychogeriatric units across the four countries, their head physicians were asked to provide data on their clinical, teaching, and research activity, as well as staff composition. Moreover, the attitudes of head physicians to current needs and future service development were explored.

**Results:**

A total of 15 psychogeriatric units were identified (3 in Bulgaria, 8 in Greece, and 4 in North Macedonia). Results show wide variation regarding the location, team size and composition, service availability, numbers of patients attending, and inpatient treatment length. Most head physicians underscored the urgent need for breakthroughs in the graduate and postgraduate education in psychogeriatrics of medical and nonmedical professionals, as well as in the interconnection of their units with community primary healthcare services and long-term care facilities for seniors via telemedicine. They would welcome the development of national standards for psychogeriatric units, potentially embodying clear pointers for action. A number of head physicians advocated the development of nationwide old age mental health registries.

**Conclusions:**

Regional disparities in resources and services for seniors’ mental health services were unveiled. These data may enrich the dialogue on optimizing psychogeriatric services through planning future cross-border collaborations mainly based on telemedicine services, especially in the era of the novel coronavirus pandemic, and training/education in psychogeriatrics of mental health professionals.

## Introduction

As the population worldwide grows and ages, the number of seniors affected by mental disorders is expected to expand in the next decades [1]. In the Balkan Peninsula, this trend is further intensified by emigration and the exodus of skilled, educated, and talented young people [2,3]. Ιn Greece, the oldest European Union (EU) member state of the region, the share of population aged 65 or older has recently risen above 21% and is expected to surpass 32% by 2050. In Albania and North Macedonia, the EU accession talks of which have recently begun, the ratio of elderly individuals to the number of persons in the working ages (old-age dependency ratio) is expected to surpass 43 and 45%, respectively, in 2050, while in both countries it did not exceed 23.5% in 2019 [4]. Over 20% of elderly people suffer from a mental or neurological disorder [5]. Such disorders result in more than 6% of all disability observed in seniors and in more than 17% of years lived with disability [5,6]. In addition, social distancing, an effective measure to decelerate the spread of the coronavirus disease 2019 (COVID-19) [7], is supposed to perplex further the situation, since social isolation pertains to high risk for depression, anxiety, and suicidality [8,9]. Interestingly, psychosocial factors, such as ageism, contribute to the high vulnerability of seniors to COVID-19 disease [10,11]. In the new terrain of the COVID-19 pandemic, the implementation of telepsychiatry for direct care and psychotherapy is a pragmatic strategy to meet the needs of elderly individuals with mental disorders [12].

Old age mental health services are more effective in looking after elderly people suffering from mental disorders in comparison to general adult psychiatric services. They are dedicated to people in later life living with (a) a recurrent, persistent, or chronic mental illness, (b) a late-onset mental illness, (c) behavioral and psychological symptoms related to neurocognitive disorders, or (d) old age medical diseases linked to psychiatric symptoms, for example, chronic obstructive lung disease [13–20]. Of note, elderly individuals, suffering from mental disorders, treated at psychogeriatric units, have less unmet needs (e.g. medication management, physical healthcare, domestic management) in comparison to elderly patients in contact to general adult mental healthcare services [21,22], because at psychogeriatric units the necessary bespoke competencies are available, so that even mild forms of old age mental disorders are recognized and properly treated, maintenance treatment is properly provided [23], and the required careful drug dose adjustment and monitoring are guaranteed [21].

Steps toward meeting the specific mental health needs of elderly people in Southern Balkans, the part of Europe that includes Albania, Bulgaria, Greece, and North Macedonia, seem to be inevitable and pressing. Healthcare services in this region are scourged by long-lasting deep socioeconomic crises, recession, and austerity policies that have impeded the adjustment of the national healthcare systems to the increasing demand for services for older adults [24–28].

The present survey aimed to increase the knowledge base of the typology, resourcing, geospatial distribution, clinical, research, and didactic activity of psychogeriatric units in Southern Balkans. In addition, it aimed to serve as an awakening voice depicting the difficulties and needs of the units’ staff. Such a base may trigger the planning of cross-border mental healthcare collaborations focused, for instance, on education, health information exchange, and cross-border coordination of services, so that in the near future the specific and distinct needs of seniors with mental disorders are adequately met and the provided old age mental healthcare in the region is harmonized [29], particularly in the light of the European Union enlargement and cohesion policies.

## Methods

Advocacy bodies of psychiatrists and old age psychiatrists in Albania (Psychiatry Division of the Tirana Medical University), Bulgaria (Ministry of Health Expert Board on mental health issues), Greece (Hellenic Psychogeriatric Association), and North Macedonia (Macedonian Psychiatric Association) were contacted and asked to provide information on existing old age mental healthcare services. Afterward, the head physicians of the identified psychogeriatric units were contacted and asked to provide data on staffing profile and composition, employed diagnostic rating instruments, numbers of patients assessed/treated annually, research, and didactic activity. In line with a previous survey on memory clinics [30], head physicians were asked to respond to three open-ended questions about: (a) their views on what is needed on the national level so that their psychogeriatric units are supported; (b) their views on the advantages and disadvantages of developing nationwide standards for the operation and certification of psychogeriatric units by Ministries of Health; and on (c) how they would use an unlimited budget for the development of their psychogeriatric unit. Data were collected between May 2019 and January 2020. After description and explanation of the aims and objectives of the survey, all head physicians agreed to participate. They replied by email to the questions and were blind to the responses of the rest of head physicians. The study did not require ethical approval.

Differences in old age mental health services between the major socioeconomic (d’unités territoriales statistiques category 1, NUTS 1) regions of Southern Balkans were studied based on the DESDE-LTC tool (Description and Evaluation of Services and Directories in Europe for Long Term) [31] and on regional sociodemographic healthcare indicators. Available in- and outpatient- and liaison psychogeriatric services were coded in Basic Stable Input of Care (BSIC), being the temporally and organizationally stable minimal unit providing coordinated care to a discrete target group of health consumers, and in different Main Types of Care (MTC). MTC taxonomy enables grouping of similarities of care delivery and distinguishing them from other care types. BSIC and MTC gross numbers and rates per 100,000 (100k) elderly inhabitants, availability of outpatient psychogeriatric services in hours/week and in hours/week/100k elderly inhabitants, placement capacity as gross number, and as rate per 100k elderly inhabitants are reported. The selection of sociodemographic healthcare indicators relied on the European Socio-demographic Schedule for the description of mental health areas through demographic characteristics related to mental disorders, as well as on the Organization for Economic Collaboration and Development [29]. The selected indicators were: (a) population aged 65 and over, (b) % of residents aged 65 and over, (c) old dependency ratio (the ratio between the number of persons aged 65 and over and the number of persons aged between 15 and 64), (d) % of residents aged 65 and over with self-perception of their health as good or very good. Data were downloaded from the Eurostat website (https://ec.europa.eu/eurostat/statistics-explained/index.php/Main_Page) in December 2019 and January 2020. To facilitate direct/standard comparisons, we chose to present data referring to major socioeconomic regions as a reasonable compromise between considering states as territorial units (NUTS0 level), even though they significantly differ in population size, and referring to basic regions for the application of regional policies (NUTS2 level), which are rarely characterized by scarcity of psychogeriatric services.

## Results

### The landscape of old age mental health units in Southern Balkans

#### Staffing profile and composition

**Figure 1.**
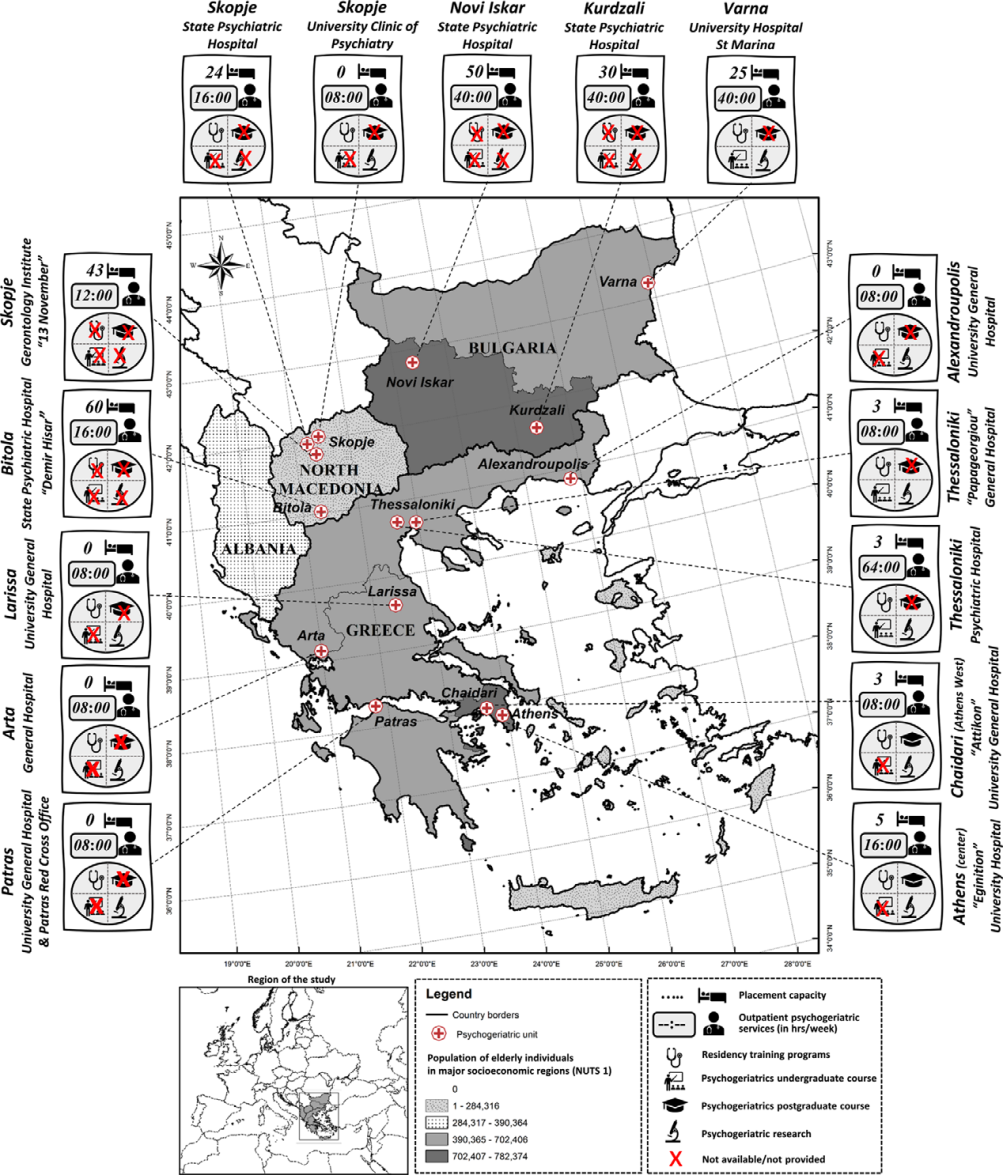
Geospatial distribution and main features of psychogeriatric units in major socioeconomic regions of Southern Balkans.

Fifteen psychogeriatric units operating in Southern Balkans were identified. All head physicians of the identified units agreed to participate in the study. The geospatial distribution, gross numbers, staffing, and clinical activity of units offering old age mental healthcare services varied across the NUTS1 regions of Southern Balkans and are succinctly depicted in Table 1 and Figure 1. The ratio of physicians to nonmedical staff at units providing both in- and outpatient services varied between 0.125 (State Psychiatric Hospital in Kurdzali) and 2 (for instance “Attikon” University General Hospital in Athens West). At psychogeriatric units that do not provide inpatient services, the ratio varied between 0.5 (University Clinic of Psychiatry Skopje) and 2 (Alexandroupolis University General Hospital).

**Table 1.** Location, staffing, didactic activities, rating tools and clinical focus of psychogeriatric units across major socioeconomic regions of Southern Balkans.

Psychogeriatric Unit		Services	Staffing		Didactic activities	Assessment scales employed			Clinical focus	
Site										
			Psychiatrist/ Non-consultant doctor	Non-medical health professionals		Psychiatric symptoms	Cognitive symptoms	Activities of daily living (ADL)	Patients assessed/treated annually (N outpatients/N inpatients)	Common diagnoses
**Albania**										
**Bulgaria**										
***South-Western and South-Central Bulgaria***										
Novi Iskar	State Psychiatric Hospital	IP, OP, L	2/1	Nurses (7), social worker (1), psychologist (1)	-	GDS, MADRS, PANSS, MADRS, YMRS, GAF	MMSE, MINI-COG test, MoCA	BADLs, ADCS-ADL	85/320	Neurocognitive disorders and delirium (50%), depression (40%)
Kurdzali	State Psychiatric Hospital	IP, OP	1/0	Nurses (8)	-	GDS, PANSS, MADRS, YMRS	MMSE, MINI-COG test, MoCA	BADLs, ADCS-ADL	70/280	Neurocognitive disorders and delirium (50%), depression (40%)
***Northern and Eastern Bulgaria***										
Varna	University Hospital St Marina	IP, OP, L	3/2	Nurses (9), psychologist (1)	PEUGC, PRTP, NRTP, GPRTP	GDS, PANSS, MADRS, HAM-D, HAM-A, NPI	MMSE; WCST, Trail making test, DSBT, Tower of Hanoi	-	100/250	Neurocognitive disorders (25%), depression (65 %),
**Greece**										
***Attica***										
Athens center	“Eginition” University Hospital	IP, OP, L	1/1*	social worker (1)	PPGC, PRTP, NRTP, GPRTP	GDS, NPI	MMSE, 3MS, MINI-COG test	BADLs, ADCS-ADL	380/41	Neurocognitive disorders (50%), mood disorders (38%)
Athens west	“Attikon” University General Hospital	IP, OP, L	1/1	psychologist (1)	PPGC, PRTP, NRTP, GPRTP	HRSD, PANSS, YMRS, SBQ-R,	3MS	IADLs	447/48	Neurocognitive disorders (6%), mood disorders (46%), psychotic disorders (18%), anxiety disorders (18%),
***Northern Greece (Epirus, Makedonia and Thrace)***										
Thessaloniki	“Papageorgiou” General Hospital	IP, OP	1/1	psychologist (1)	PEUGC, PRTP, NRTP, GPRTP	GDS, HRSD, NPI	MMSE, Clock test, SIB, WCST, Trail making test, DSBT, Verbal fluency tests, Tower of Hanoi, CDR	IADLs	700/120	Neurocognitive disorders (40%), mood disorders (60%),
Thessaloniki	Psychiatric Hospital of Thessaloniki	IP, OP	3/0	Nurses (2), psychologist (1), music therapist (1)	PEUGC, PRTP, NRTP, GPRTP	GDS, HRSD, NPI	MMSE, Clock test, SIB, WCST, Trail making test, DSBT, Verbal fluency tests, BNT, Tower of Hanoi, CDR	IADLs	1400/100	Neurocognitive disorders (30%), depression and anxiety disorders (30%), psychotic disorders(30%)
Alexandroupolis	Alexandroupolis University General Hospital	OP, L	1 /1*	Nurse (1)	PRTP, NRTP, GPRTP	GDS	MMSE, Verbal fluency tests, Clock test		180/0	Neurocognitive disorders (30%), mood disorders (70%)
Arta	Arta General Hospital	OP, L	1/0	psychologist (1)	PRTP	GDS, CSDD	MMSE, MoCA, 3MS	BADLs FAQ	110/0	Neurocognitive disorders (37%), mood disorders (38%), anxiety disorders (15%), psychotic disorders (10%)
***Central Greece (Thessaly, Ionian islands, Western Greece, Peloponnese, Region of Central Greece)***										
Larissa	Larissa University General Hospital	OP, L	2/0	Nurse (1), psychologist (1)	PRTP, NRTP, GPRTP	GDS, AES, HARS, NPI, CSDD	MoCA, CAMCOG, MMSE, MINI-COG test	ADCS-ADL, IADLs	127/0	Neurocognitive disorders (40%), mood disorders (60%),
Patras	Patras University General Hospital and Patras Red Cross Office	OP	1/1	Nurse (1)	PRTP, NRTP, GPRTP	GDS, NPI	ACE III, MoCA, Rey- Osterrieth Complex Figure, FAB	BADL	246/0	Neurocognitive disorders (35%), mood disorders (65%)
***Aegean islands and Crete***										
**North Macedonia**										
Skopje	State Psychiatric Hospital	IP, O	2/0	Nurses (7), social worker (1), psychologist (1)	PRTP	GDS, HRSD, PANSS, YMRS	MMSE, MINI-COG, MoCA, ACE-R, RAVL-T, BNT	ADCS-ADL	1000/80	Neurocognitive disorders (60%) depression (20%)
Skopje	University Clinic of Psychiatry	OP	1/0	Nurse (1), psychologist (1)	PRTP	GDS, HRSD, PANSS, YMRS	MMSE, MINI-COG, MoCA, ACE-R, RAVL-T, BNT	ADCS-ADL	225/0	Neurocognitive disorders (75%), depression (20%)
Skopje	Gerontology Institute “13 November”	IP, OP, L	2/0	Nurses (4), social worker (1), psychologist (1)		GDS, HRSD, PANSS	MMSE, MINI-COG, MoCA	ADCS-ADL	700/50	Neurocognitive disorders (80%), depression (20%)
Bitola	State Psychiatric Hospital Demir Hisar	IP, OP	2/0	Nurses (5) social worker (1), psychologist (1)		GDS, HRSD, PANSS	MMSE, MINI-COG, MoCA	ADCS-ADL	800/ 20	Neurocognitive disorders (50%), depression (20%)

* General practiotioner

ACE III: Addenbrooke’s cognitive examination III; ADCS-ADL: Alzheimer’s disease cooperative study group activities of daily living inventory; AES: apathy evaluation scale; BADLs: Bristol activities of daily living scale; BNT: Boston naming test; CAMCOG: Cambridge cognition examination; CDR: Clinical dementia rating; CSDD: Cornell scale for depression in dementia; DST: Digit span tasks; FAB: Frontal assessment battery; FAQ: Functional activities questionnaire; GDS: geriatric depression scale; GPRTP: general practice residency training program; HARS: Hamilton anxiety rating scale; HRSD: Hamilton rating scale for depression; IADL: Lawton instrumental activities of daily living scale; IP: inpatient services; L: liaison services; MMSE: Mini mental state examination; 3MS: Modified mini-mental state; MoCA: Montreal cognitive assessment; NPI: neuropsychiatric inventory; NRTP: neurology residency training program; OP: outpatient services; PANSS: Positive and negative syndrome scale; PPGC: psychogeriatrics postgraduate course; PRTP: psychiatry residency training program.

#### Clinical activity

The gross numbers of patients assessed/treated annually and the established diagnoses varied across the units, too (Table 1). The ratio of elderly out- to inpatients assessed/treated annually began from 0.20 (State Psychiatric Hospital in Kurdzali) and rocketed to 40 in the case of the psychogeriatric unit at the State Psychiatric Hospital Demir Hisar in Bitola, even though the number of beds per psychogeriatric unit reached its maximum at this unit. The ratio of annually treated inpatients to available psychogeriatric beds was lowest in Bitola (0.33) and rocketed to 10 at the “Papageorgiou” General Hospital in Thessaloniki. Seven units provide psychogeriatric liaison services, which cover in- and outpatients of other hospital departments, memory clinics, memory day centers, and nursing homes. Neurocognitive and mood disorders counted for at least 60% of the annually established diagnoses except for the psychogeriatric unit at the “Attikon” University General Hospital in Athens West, where only 6% of patients suffered from neurocognitive disorders. Except for one unit in the region of Northern and Eastern Bulgaria, at which patients’ functional performance is not routinely assessed, all other units employ valid rating instruments for assessing the triad of symptom groups of such disorders, i.e. cognitive dysfunction, difficulties in activities of daily living, and psychiatric symptoms.

#### Research and didactic activity

Data on research conduct and didactic activities unveiled a wide variation (Figure 1). Research projects with special focus on old age mental health issues mainly focus on dementia behavioral and psychological symptoms and sensory impairment, affective disorders and suicidal behavior in seniors, prevention and treatment of postoperative delirium, liaison, and telepsychogeriatrics. Besides providing bedside teaching to undergraduate students as part of their rotation at university departments of psychiatry, most of the psychogeriatric units provide training for residents of psychiatry and in many cases of neurology, as well as general practitioners. Of note, the curricula of the Faculties of Medicine in Varna and Thessaloniki include elective undergraduate courses on psychogeriatrics. The only psychogeriatrics postgraduate course in Southern Balkans is offered by the National and Kapodistrian University of Athens, and it covers a wide spectrum of aging and old age health issues as indicated by its title “Physiology of Aging and Geriatric Syndromes.”

#### Regional disparities of old age mental healthcare services

Sociodemographic characteristics, placement capacity, and availability of outpatient services varied across the major sociodemographic regions of Southern Balkans (Table 2 and Figure 1). The availability of outpatient psychogeriatric services was highest in North Macedonia and lowest in Central Greece, while no such services were available at all in Albania and in Aegean islands and Crete. The picture of the placement capacity rates per 100 k seniors followed a similar pattern. The placement capacity rate per 100 k seniors was 10.23 in South-Western and South-Central Bulgaria and only 1.04 in Attica, even though the elderly population difference between the two regions did not exceed 16,100 seniors. Of note, the relative abundance of mental health services for elderly individuals in North Macedonia does not seem to be reflected in health self-perception as good, since only 29.3% of elderly individuals in the country perceived their health at least as good. This indicator, which is, however, not exclusively contingent on mental health issues, was in the case of North Macedonia lower compared to Greece and only slightly higher than in Bulgaria (40.5 and 24%, respectively). Regarding the relationship between out- and inpatient services availability (Table 2), in North Macedonia, the ratio of out- to inpatient old-age mental health services reached its minimum (0.41), and in Northern Greece reached its maximum (13.26).

**Table 2.** Sociodemographic data, availability, placement capacity, and diversity of psychogeriatric services in major socioeconomic regions of Southern Balkans.

	Albania	Bulgaria		Greece				North Macedonia
		*South-Western and South-Central Bulgaria*	*Northern and Eastern Bulgaria*	*Northern Greece*	*Central Greece*	*Attica*	*Aegean islands and Crete*	
NUTS1 code	AL0	BG4	BG3	EL5	EL6	EL3	EL4	MK0
Population aged 65 and over	390364	782374	698112	702406	638840	766316	229990	284316
% of residents aged 65 and over	13.6	22.2	19.8	22.8	23.5	20.4	19.4	13.7
Old dependency ratio	19.7	35	30	36.2	37.6	31.4	30.2	19.5
% of residents aged 65 and over with self-perception of their health as good or very good		24		40.5				29.3
Availability of BSICs	0	2	1	4	2	2	0	4
BSICs availability per 100k elderly inhabitants	0	0.25	0.14	0.57	0.31	0.26	0	1.41
Availability of MTCs	0	4	3	6	3	6	0	8
MTCs availability per 100 k elderly inhabitants	0	0.51	0.43	0.85	0.47	0.78	0	2.81
Αvailability of outpatient psychogeriatric services in hours/week	0	80	40	80	16	24	0	52
Availability of outpatient psychogeriatric services in hours/week per 100k elderly inhabitants	0	10.23	5.73	11.40	2.50	3.13	0	18.29
Placement capacity	0	80	25	6	0	8	0	127
Placement capacity per 100 k elderly inhabitants	0	10.23	3.58	0.86	0	1.04	0	44.67

NUTS1 code: Code of a major socioeconomic region of the studied countries according to the nomenclature of territorial units for statistics; Old-age dependency ratio: This indicator is the ratio between the number of persons aged 65 and over (age when they are generally economically inactive) and the number of persons aged between 15 and 64. The value is expressed per 100 persons of working age (15–64).

Abbreviations: BSIC, Basic stable input of care, being the temporally and organizationally stable minimal unit providing coordinated care to a discrete target group of health consumers; MTC, Main type of care, being the descriptor of the basic activity carried out in one BSIC; Placement capacity.

*Source:* eurostat.

### Current needs and future perspectives of psychogeriatric services

#### Current needs

Responding to the question about what is most urgently needed nationally so that the operation of psychogeriatric services is facilitated and further developed, all head physicians of psychogeriatric units in North Macedonia and two in Bulgaria emphasized the urgent need for developing nationwide standards for psychogeriatric care, while in Greece, head physicians put on the top of such an agenda the acknowledgment of psychogeriatrics as a subspecialty and the inclusion of psychogeriatrics in training programs for medical and nonmedical mental health professionals. In addition, most of the head physicians underscored the importance of educational and clinical interconnections of primary healthcare services with the local psychogeriatric unit(s). Head physicians in Bulgaria opted for increasing the ratio of physicians to nonmedical staff of psychogeriatric units to at least 0.33, the lowest value of which is now 0.125.

#### Advantages and disadvantages of developing nationwide standards for psychogeriatric units

In response to the advantages/disadvantages of developing nationwide standards for the operation and certification of psychogeriatric units by Ministries of Health, offering to seniors with mental disorders standardized high-quality mental health services was mentioned as the main advantage, while the importance of such a development for demarcating the role of old age psychiatry in the care of seniors was also underlined. Nonetheless, the point was raised by the majority of head physicians that in the light of current insufficient financial resources and the features of a number of psychiatric hospitals (e.g., low patients’ annual turnover per hospital ward) a possible dogmatic implementation of such standards may hamper the sustainability of the already operating psychogeriatric units and prevent hospital boards from inaugurating new ones.

#### Unlimited budget and future development of old age mental healthcare services

Finally, all head physicians reckoned that they would use resources to build multidisciplinary teams providing in-, outpatient, and liaison services and create and expand interconnected networks of different stakeholders of old age mental healthcare. Five head physicians would support the development of telepsychogeriatric networks, even though the regulatory legal framework for telepsychiatry still needs to be clarified as they underlined. Moreover, most head physicians would also invest in the specialized training on psychogeriatrics of physicians and of wider health and social care workforce. Interestingly, psychogeriatrics is understood by seven head physicians as part even of undergraduate curricula for both medical and nonmedical professionals. Three heads would use an unlimited budget to develop nationwide old age mental health registries.

## Discussion

The rapid population of aging in Southern Balkans has created a new environment of needs for the management of old age mental disorders. Relying on the DESDE-LTC tool and on regional sociodemographic health care indicators, the findings of the present study enable standard comparisons and may be easily considered/incorporated in future studies and meta-analyses [29,32]. Our observations point to the gross regional disparities in seniors’ mental health care services in this part of Europe. Most of the psychogeriatric units are located in large cities or are based at psychiatric hospitals, while in peripheral cities or in whole major socioeconomic regions like the region of Aegean islands and Crete or even in whole countries like Albania no such services are available. Since old age mental health services availability does not follow the pattern of variation of the population of elderly people across the studied major socioeconomic regions, it becomes clear that the development of such services was not based on mapping or top-down planning of service provision. Such imbalances have been observed in geriatric mental health services in other European countries too [33], as well as in memory clinics [30]. Despite these imbalances and the international trends of developing specialized old age mental health care services [6,13,15], new psychogeriatric units in the studied region are being planned and inaugurated in a relatively slow rhythm. This reluctance can be attributed to a certain extent to the negative effects of the deep socioeconomic crises and dearth of financial resources that have scourged the region of Southern Balkans.

Strategies to address the issue of regional imbalance of available old age mental health services are urgently needed. In the short run, telepsychogeriatrics may embody a pragmatic strategy, especially in the light of the high vulnerability of elderly people to the devastating effects of the COVID19 pandemic and the importance of social distancing and seniors’ mobility reduction as measures to combat COVID19 spread [9,12,34,35]. Medical and nonmedical professionals in peripheral cities and towns could be supervised and supported via telemedicine by experts in psychogeriatrics in making diagnostic and therapeutic decisions in more or less complex cases [36]. The telepsychogeriatrics model of care for patients in remote areas (i.e. the island of Andros in the region of Aegean islands and Crete, Greece, where no psychogeriatric units operate) embodies such an intervention [37]. However, the legal regulatory framework needs to be clarified, so that risks pertaining to telemedicine, such as privacy concerns or security breaches, are minimized [38]. Designing and implementing transnational telepsychogeriatrics networks in cooperation with the telemedicine startup ecosystem may catalyze the incorporation of telepsychiatry in psychogeriatric services in Southern Balkans, since such projects familiarize both health care providers and patients with the practicability and efficiency of telemedicine [39]. European Union cohesion and integration funds could be sources of financial support of such networks.

The issue of the regional imbalance of available old age mental health services should be addressed more drastically in the long run. The development of nationwide old age mental health registries is a way to estimate reliably the prevalence and incidence of old age mental disorders in different socioeconomic regions of Southern Balkans, as well as to grasp where such disorders are diagnosed (for instance, at primary health care centers or at specialist hospital clinics not related to psychogeriatric units). The diversity of local contexts and resources, existing practices, and relationships will be brought into light, so that comprehensive and efficient services meeting international standards and responding to the local needs of elderly people are developed. Moreover, taking into account the intense nature of cross-border mobility between the countries of Southern Balkans [40–42], collecting such data could form, in the long run, the bedrock for developing sustainable cross-border mental health care collaborations not excluding from their focus seniors living in peripheral cities and towns.

The psychogeriatric units in Southern Balkans are characterized by variability with regard to staffing. The ratio of physicians to nonmedical health professionals varied across the units, while only three units had a social worker, and none had an occupational therapist, even though occupational therapy belongs to the most well-studied non-pharmacological treatments of old age mental disorders [30,43–45]. On the other hand, psychologists enriched the teams of eight psychogeriatric units and safeguard the application of psychotherapeutic strategies. However, it remains unclear, if they had undergone special training and were experienced in psychotherapeutic interventions suitable for seniors with mental disorders (e.g. problem-solving therapy, reminiscence therapy) [46]. The relatively limited multiprofessional character of the psychogeriatric teams can lead to disproportionately high employment of pharmacological interventions resulting commonly in polypharmacy and high medication doses associated with severe or even life-threatening side effects, as well as high costs [47–50]. Therefore, pragmatic solutions are urgently needed. Again, telepsychogeriatric networks may partially compensate for the absence of a specialized professional at a particular psychogeriatric unit in the short run, while education and training programs in psychogeriatrics seem to form the long-term solution.

Even though specific training in psychogeriatrics has been placed at the top of the list of priorities of the plans of action of psychogeriatric associations across the globe and was seen as top priority by many head physicians of psychogeriatric units in Southern Balkans [33,51], educational psychogeriatric programs in this part of Europe are still in their infancy. The undergraduate psychogeriatrics elective courses at the University of Varna and at the Aristotle University of Thessaloniki and the “Physiology of Aging and Geriatric Syndromes” postgraduate program at the National and Kapodistrian University of Athens embody three pioneer efforts. In addition, in Greece, mandatory specific training in geriatric psychiatry with a minimum duration of 4 weeks and a maximum of 3 months is intended to become part of the general psychiatry residency program. This change may be interpreted as a first, timid step toward recognizing old age psychiatry as a subspecialty. Nonetheless, staff training in psychogeriatrics would remain incomplete, if medical geriatric psychiatry training is not coupled with adequate educational programs for nonmedical professionals who work with seniors [6]. Specific training in psychogeriatrics for all health and social care professionals can enhance their valuable contribution to improving the patients’ emotional, intellectual, and social well-being [51].

Developing standards for the operation and certification of psychogeriatric units was approached by all psychogeriatric units’ head physicians as a tool for improving health care service quality. Nonetheless, it is not a panacea. Nationwide standards can provide clear pointers on action for setting up and optimizing psychogeriatric services. For instance, they can pave the way toward psychogeriatric units providing services for both inpatients and outpatients, as well as liaison services, since there is currently a heterogeneity regarding the range of services provided by the different psychogeriatric units. In addition, they can foster the deepening of deinstitutionalization (e.g. reduction of the length of inpatient treatment) and the setting up of community-based old age mental health services. It is noteworthy that in a number of countries the development of standards for the operation of memory clinics was seen as a pressing issue [52,53]. Nevertheless, the lack of clarity of the goals and outcomes of each memory clinic undermined the efforts to assess care quality of each particular center [30]. Thus, such standards should not embody dogmatic canons threatening the existence of currently operating psychogeriatric units, which form in many cases a well-balanced compromise between local needs and available funds and resources, or discouraging attempts to set up new ones.

Several limitations should be taken into account when interpreting the findings of the present survey. First, it exclusively focused on psychogeriatric services and did not cover either neurological or geriatric services, which may compensate in many cases for the regional dearth of psychogeriatric services. Nevertheless, the aim of our study was to provide an overview of psychogeriatric services in Southern Balkans, which are more effective in meeting the specific care needs of elderly people suffering from mental disorders compared to other types of health care services [21]. Second, the borders of major socioeconomic regions are permeable and it cannot be precluded that in a number of cases the transregional mobility for health reasons is more dense than intraregional mobility. Considering such uncertainties may be a future research topic. Third, the study of the current needs and future directions of psychogeriatric units relied on responses of head physicians, while the attitudes of nonmedical professionals were not directly grasped. It was assumed that head physicians objectively reflected the needs and difficulties of their entire team and accurately depicted plans and changes being in the pipeline. Fourth, posing open-ended questions in written form and expecting answers via email may have not given head physicians the necessary space to adequately express themselves. However, in the absence of few, clear-cut, and easily predictable answers, written responses to open-ended questions embody a reasonable compromise between relying on multiple-choice questions and interviews over the phone, the interpretative endeavors of which may have embodied an additional source of bias [54].

In conclusion, long-lasting and painstaking efforts of psychiatrists with special interest in psychogeriatrics have resulted in a network of 15 psychogeriatric units in Southern Balkans, which strive to offer services according to international standards and to familiarize students and residents with the challenges of psychogeriatrics. Despite the progress made in the last years, further steps concerning training/education in psychogeriatrics, the struggle against the gross regional disparities in resources and services and, last but not least, the development of old age mental health registries are urgently warranted, so that the local needs of elderly people with mental disorders are adequately met.

## Data Availability

The datasets used and analyzed during the current study are available from the corresponding author on reasonable request.
